# Comparison of safety and efficiency of microendoscopic discectomy with automatic nerve retractor and with nerve hook

**DOI:** 10.1093/rb/rbw029

**Published:** 2016-09-27

**Authors:** He-Ping Yin, Yu-Peng Wang, Zhi-Ye Qiu, Zhi-Cai Du, Yi-Min Wu, Shu-Wen Li

**Affiliations:** ^1^The Second Affiliated Hospital, Inner Mongolia Medical University, Hohhot 010030, China; ^2^Institute of Regenerative Medical Materials, School of Materials Science and Engineering, Tsinghua University, Beijing 100084, China

**Keywords:** minimally invasive lumbar discectomy, automatic nerve retractor, microendoscopic discectomy, nerve hook

## Abstract

This study compares the safety and efficiency of two techniques in microendoscopic discectomy (MED) for lumbar disc herniation. The two techniques are MED with automatic nerve retractor and MED with nerve hook which had been widely used for many years. The former involves a newly developed MED device which contains three parts to protect nerve roots during operation. Four hundred and twenty-eight patients underwent MED treatments between October 2010 and September 2015 were recruited and randomized to either intraoperative utilization of automatic nerve retractor (*n* = 315, group A) or application of nerve hook during surgery (*n* = 113, group B). Operation time and intraoperative bleeding volume were evaluated. Simultaneously, Visual Analogue Scales (VAS) and muscle strength grading were performed preoperatively, and 1, 2, 3 days, 1, 2 weeks, 3 and 6 months postoperatively. No dramatic difference of pain intensity was observed between the two groups before surgery and 6 months after surgery (*P* > 0.05). The operation time was shorter in group A (30.30 ± 1.89 min) than that in group B (59.41 ± 3.25 min). Group A (67.83 ± 13.14 ml) experienced a significant decrease in the amount of blood loss volume when compared with group B (100.04 ± 15.10 ml). There were remarkable differences of VAS score and muscle strength grading after postoperative 1, 2, 3 days, 1, 2 weeks and 3 months between both groups (*P* ≤ 0.05). MED with automatic nerve retractor effectively shortened operation time, decreased the amount of bleeding, down-regulated the incidence of nerve traction injury.

## Introduction

Discectomy symptomatic lumbar disc herniation has been widely used in spinal surgical operation. Minimally invasive techniques evolved where paraspinal muscular elevation is done for only 2–3 cm by using specialized parts [1–3], such as microendoscopic discectomy (MED) with nerve hook. Minimally invasive techniques have the theoretical advantage of less tissue scarring and better visualization of the dural, roots and disc space, and hence are expected to have better postoperation outcomes.

**Figure 1.**
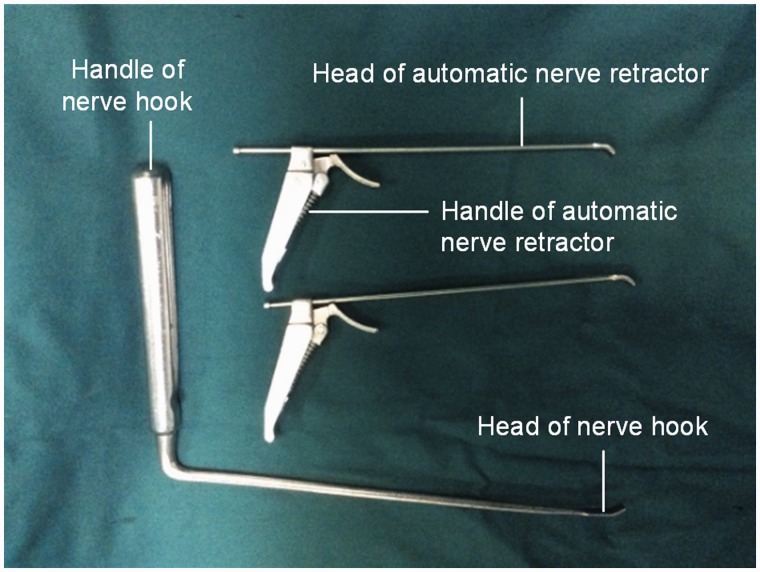
The photos of automatic nerve retractor and never hook

MED with nerve hook is an effective method for treatment of lumbar disc herniation in clinical practice because of its smaller trauma, less bleeding, full decompression, rapid postoperative recovery, etc. [4–9]. However, this MED operation includes the complex operation, such as opening the window decompression, the electric coagulation, the removal of the flavum ligament, the dissection and protection of dural sac and nerve root, the protrusion of the intervertebral disc and the expansion of the spinal canal [10–12]. It is therefore difficult to finish such sophisticated and detailed operation process in high quality within a shorter time in a diameter 16–18 mm working channel. It is bound to prolong the operation time, cause a transient nerve root traction injury, and even result in the small blood vessels inner spinal canal bleeding [12, 13]. In addition, the nerve hook’s head with sharp corners is easy to damage the nerve roots and dural sac, so the risk of the operation will be elevated. To compensate for these shortcomings, the automatic nerve retractor was invented [14]. It is shown in Fig. 1 that the photos of automatic nerve retractor and nerve hook.

## Materials and methods

### The device

Automatic nerve retractor is composed of three parts: detacher, handle and a fixed needle, and the material is 304 medical stainless steel. Detacher part is resembling the general never hook, but the different from the nerve hook is the hollow structure (inner diameter = 1.6 mm, length = 145 mm, width (positive) = 3.0 mm, thicken (later) = 2.5 mm; head end is 30° arc), which includes two specifications: the width of 3.5 and 5 mm. Its function is to peel, stretch and protect the dural sac and nerve root, and expose the prolapsed intervertebral disc. The rear of detacher can be connected with the suction device to suck blood and smoke produced by electrocoagulation during surgery to ensure a clear operative vision. The handle portion (length = 60 mm) is composed of a gear wrench, a spring and a handle, which is connected with the detacher and can be catered the needs to move up and down. While the position is satisfied, you can fix the locker on the working retractor, to effectively maintain the protection of the dural sac and nerve root and the exposure of the herniated disc. The fixed needle length is 155 mm, and diameter is 1.2 mm. The rear portion of it is a cap-like structure. Use the fixed needle through detacher rear insertion, the hollow body, the head end finally pulled out, which fixed automatic nerve retractor and prevent the movement of the detacher.

### General information

#### Retrospective analysis

428 cases (male 225 cases, female 203 cases, aged from 13 to 88 years, average 46 years old. L_1/2_ discs 4 cases, L_2/3_ discs 7cases, L_3/4_ discs 36 cases, L_4/5_ discs 213 cases, L_5_/S_1_ discs 168 cases) who treated with MED in our hospital from September 2010 to October 2015 were enrolled. Prominent type: Central 149 cases, lateral 172 cases, extreme lateral 21 cases, ruptured and free 86 cases. Thirty-nine of the cases with calcification, and 226 cases of vertebral canal stenosis. X-ray lateral anteroposterior and flexion-extension stress lateral, CT (computed tomography), MRI (magnetic resonance imaging) and EMG (electromyography) were examined before surgery.

### Operation methods

**Figure 2.**
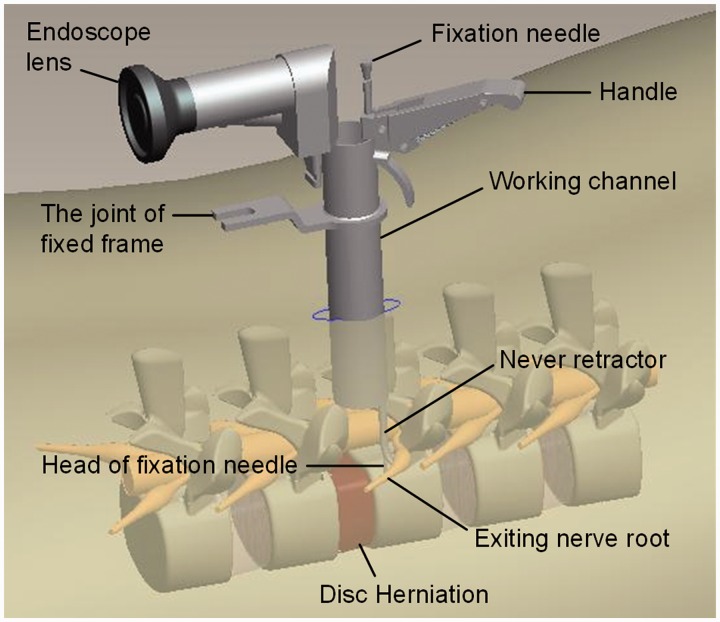
The diagram of MED with automatic nerve retractor

**Figure 3.**
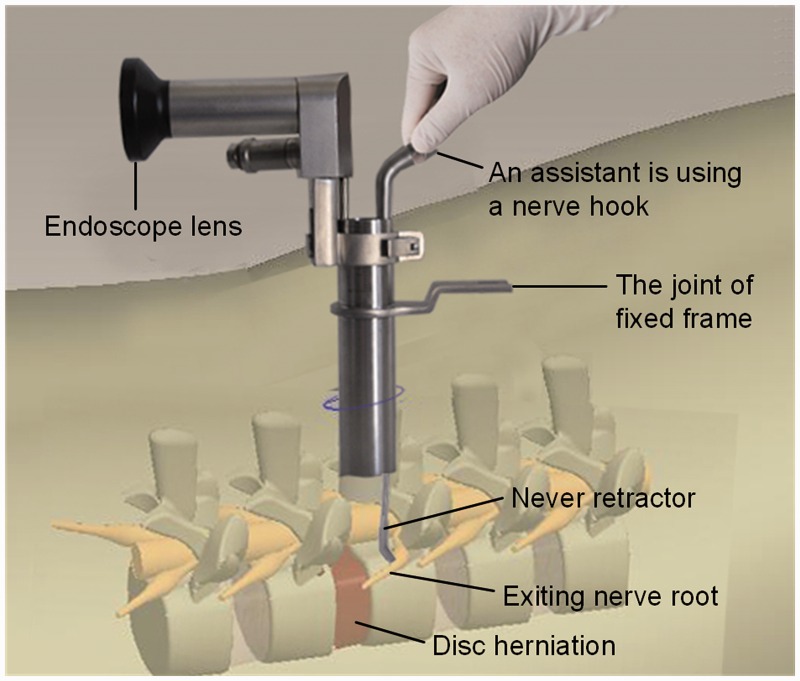
The diagram of MED with nerve hook

The patient was placed prone on an operating table that will accommodate fluoroscopy after adequate anesthesia. In general, hip flexion 45° and knees flexion 45° position that provides lumbar kyphosis to conduce depression during surgery. Then regular disinfection and drape, next to the spinous process of the symptomatic side 8-mm percutaneous insert K-wire up to the inferior edge of the lamina. Using “C-shaped” X-ray machine fluoroscopic verification the placed, then draw the K-wire, and applied a 11# blade to make an ∼2-cm incision (depending the needed exposure), directly access to the lamina and facet joint surface (index finger touch), the increasing dilating tube inserted to the surface of ligamentum flavum, thus a 1.6-cm diameter working channel was set up. Then fluoroscopy to verify the position and trajectory. A little bite under the mirror and upper and lower lamina and facet edge and ligamentum flavum hypertrophy removal, exposed the dural sac and nerve root symptom side. The first automatic nerve retractor (group A) was placed into working tube under endoscopic imagine 64 times as truth frame, using the end of automatic nerve retractor head retracted the dural sac to the head side of spinal, along the axis of stripped body and move the handle to the appropriate position, locked and fixed on the casing margin. Similarly, the second automatic nerve retractor was placed into the casing, then distract the nerve root(s) which lead to clinical symptoms. Simultaneously, the lumbar intervertebral disc protrusion was fully exposed. Then the protrusion will be removed routinely. Drainage Strip or negative pressure drainage tube was putting into deep of the skin incision (Fig. 2). While B group finished above steps that reveal and retract nerve root(s) by one assistant using nerve hook during operation, other steps were same as group A (Fig. 3).

The pictures below vividly depict the status of MED with automatic nerve retractor (Fig. 2) and nerve hook respectively (Fig. 3). The fixation needle of automatic nerve retractor has been inserted vertebral 4–5-mm length to protect the nerve roots from a transient traction injury and maintain constant force while an assistant is applying a nerve hook to retract exiting nerve root without any fixing device.

### Evaluation methods

Comparison of operation time and intraoperative blood loss volume were conducted with both groups A and B. Visual Analogue Scales (VAS) and muscle strength grading were performed preoperative, postoperative 1, 2, 3 days, 1, 2 weeks, 3 and 6 months. The pain intensity was documented by VAS [15] and the changes of lower limb muscle strength before and after operation were recorded by muscle strength grading according to the ASIA score [16, 17].

### Statistical methods

SPSS13.0 (SPSS Company, USA) statistical software was applied to perform data description and analysis. All data were presented as mean ± standard deviation (SD). Mann–Whitney *U*-test was used to compare the two groups of independent samples. A comparison of several relevant samples using Friedman rank sum test, and the correlation between the two groups was compared with the Wilcoxon rank sum test. Wilcoxon rank sum test was used in a comparison of the two groups of related samples. *P* ≤ 0.05 is statistically significant.

## Results

Group B exhibited delayed operation duration (59.41 ± 3.25 vs. 30.30 ± 1.89 min, *P* = 0.05) compared with group A (30.30 ± 1.89 min). In addition, the intra-operation bleeding volume of group A (67.83 ± 13.14 ml) is less than that of group B (100.04 ± 15.10 ml) (Table 1). The difference of VAS score and muscle strength grading of two groups was no statistically significance (*P* > 0.05) before surgery and at 6 months after operation, but VAS score and muscle strength grading were significant differences (*P* ≤ 0.05) (Table 2) between two groups after postoperation 1, 2, 3 days, 1, 2 weeks, 3 months.

**Table 1.** Comparison of operative time and intraoperative blood loss in groups A and B

	*n*	Operation time (min)	Intra-operation bleeding volume (ml)
Group A	315	30.30 ± 1.89	67.83 ± 13.14
Group B	113	59.41 ± 3.25	100.04 ± 15.10
Statistical value		−15.909	−14.693
*P* values		0.000	0.000

Note: the rank sum test of two independent samples

**Table 2.** Comparison of two groups’ VAS scores and muscle strength grading

	**VAS scores**		Statistics value	*P* values	**Muscle strength grading**		Statistics value	*P* values
	Group A	Group B			Group A	Group B		
*n*	315	113			315	113		
Pre-operation	8.22 ± 0.66	8.12 ± 0.65	−1.428	0.153	4.30 ± 0.49	4.36 ± 0.54	−1.168	0.243
Postoperation 1 day	7.51 ± 1.17	8.15 ± 0.92	−5.129	0.000	4.82 ± 0.39	3.41 ± 1.37	−12.954	0.000
Postoperation 2 days	6.68 ± 0.47	8.15 ± 0.92	−13.608	0.000	4.82 ± 0.39	3.41 ± 1.37	−12.954	0.000
Postoperation 3 days	5.51 ± 0.53	7.76 ± 0.50	−16.576	0.000	4.82 ± 0.39	3.41 ± 1.37	−12.954	0.000
Postoperation 1 week	4.75 ± 0.65	7.19 ± 0.48	−16.605	0.000	4.82 ± 0.39	3.53 ± 1.12	−12.954	0.000
Postoperation 2 weeks	4.75 ± 0.65	5.63 ± 0.90	−9.581	0.000	4.82 ± 0.39	3.83 ± 1.03	−12.151	0.000
Postoperation 3 months	3.87 ± 0.62	3.89 ± 0.57	−0.474	0.636	4.82 ± 0.39	4.42 ± 1.07	−3.702	0.000
Postoperation 6 months	3.80 ± 0.57	3.81 ± 0.43	−0.448	0.654	4.82 ± 0.39	4.75 ± 0.81	−0.619	0.536

Note: the rank sum test was used in two independent samples.

## Discussion

Nucleus pulposus removal was completed via posterior approach using MED with imaging system, working channel and slender delicate surgical instruments. The essential operation principle of MED is similar to open surgery principle, and the equipments of MED are also originated from the open surgical instruments. The instruments of MED, especially nerve hook and suction device, were designed slender and delicate to match the narrow working tube [18, 19]. So it was awkward to finish the operation in such a limited working space. Especially using conventional nerve hook to reveal dural sac and nerve root, exposure lumbar intervertebral disc protrusion. Therefore, it is more complicated and difficult operation when confronted with complex situation, such as huge disc herniation and spinal stenosis [20–22], during operation.

The operation ream and the instruments were similar in the two groups except intraoperative application of automatic nerve retractor in group A and utilization of nerve hook in group B. However, the results of operation time, blooding volume intraoperation displayed dramatic differences. This phenomenon illustrated that more convenient operation and more clear vision of surgery field with the application of automatic nerve retractor during MED surgery than utilization of nerve hook. VAS score and muscle strength grading were compared after postoperative 1, 2, 3 days, 1, 2 weeks, 3 months, there were significant differences (*P* ≤ 0.05). The result show that the nerve roots and dural sac were revealed and retracted by automatic nerve retractor during surgery, which can more clearly exposed intervertebral disc protrusion, and more convenient operation in the separation of pull open process according to the need to control the implementation efforts, and maintain a constant tension in order to avoid the weakness because of the assistant artificial traction appear excessive force or hard enough. Meanwhile, the application of automatic nerve retractor by locking tightly and fixed on the tube during the surgery, in this way, the protective effect of stripping ion on the dural sac and nerve root(s) can be effectively maintained, thereby reducing or no nerve traction injury. While in group B, a transient nerve injury was mostly occurred caused by the sharper head of nerve hook. After all, the nerve root edema gradually subsided, and muscle strength gradually recovered at 1 week after the operation. And with the passage of time, muscle strength was gradually renewed at 6 months after operation. In addition, the use of automatic nerve retractor can reduce an assistant. The VAS score and muscle strength grade of the two groups were no statistically significant at 6 months after surgery, which may be due to a transient nerve injury is not serious, and the original function can be restored by body itself in a few days after the operation. Although the application of automatic nerve retractor meets the current operation demands, there are still weakness. First, this device is made of metal material and hard for nerve root and dural sac during operation. Second, it lacks effective equipment to evaluate the degree of intraoperative nerve injury. Third, the spring of the handle portion may engender the decrease in locking strength when it’s weakened. Last, the suction connecting hole and a fixing hole commonly use one tube, the other function is limited due to one function was activated and utilized during surgery.

## Conclusions

The advantage of applying automatic nerve retractor during MED surgery is safer and efficiency when we face complex situation—huge disc herniation and spinal stenosis than that of utilization of nerve hook. As alternatives of nerve hook, automatic nerve retractor can bring a desirable result. In addition, utilization of automatic nerve retractor has the benefit of shorten operation time, less blood loss and reduce an assistant. So far, we have not found a case of nerve root injury or dura leakage caused by the use of automatic nerve retractor in our hospital.

## Funding

This work was in part supported by National Natural Science Foundation of China, No. 81260287, and by Regional Natural Science Foundation of Inner Mongolia of China, No. 2014MS0855.

*Conflict of interest statement*. None declared.
